# Eyes-Open and Eyes-Closed Resting States With Opposite Brain Activity in Sensorimotor and Occipital Regions: Multidimensional Evidences From Machine Learning Perspective

**DOI:** 10.3389/fnhum.2018.00422

**Published:** 2018-10-18

**Authors:** Jie Wei, Tong Chen, Chuandong Li, Guangyuan Liu, Jiang Qiu, Dongtao Wei

**Affiliations:** ^1^School of Electronic and Information Engineering, Southwest University, Chongqing, China; ^2^School of Mathematics and Statistics, Southwest University, Chongqing, China; ^3^Chongqing Key Laboratory of Nonlinear Circuit and Intelligent Information Processing, College of Electronic and Information Engineering, Southwest University, Chongqing, China; ^4^Institute of Psychology, Chinese Academy of Sciences, Beijing, China; ^5^Department of Psychology, Southwest University, Chongqing, China

**Keywords:** resting state fMRI, eyes-open, eyes-closed, fALFF, ReHo, VMHC, DC, brain activity

## Abstract

Studies have demonstrated that there are widespread significant differences in spontaneous brain activity between eyes-open (EO) and eyes-closed (EC) resting states. However, it remains largely unclear whether spontaneous brain activity is effectively related to EO and EC resting states. The amplitude, local functional concordance, inter-hemisphere functional synchronization, and network centrality of spontaneous brain activity were measured by the fraction amplitude of low frequency fluctuation (fALFF), regional homogeneity (ReHo), voxel-mirrored homotopic connectivity (VMHC) and degree centrality (DC), respectively. Using the public Eyes-open/Eyes-closed dataset, we employed the support vector machine (SVM) and bootstrap technique to establish linking models for the fALFF, ReHo, VMHC and DC dimensions. The classification accuracies of linking models are 0.72 (0.59, 0.82), 0.88 (0.79, 0.97), 0.82 (0.74, 0.91) and 0.70 (0.62, 0.79), respectively. Specifically, we observed that brain activity in the EO condition is significantly greater in attentional system areas, including the fusiform gyrus, occipital and parietal cortex, but significantly lower in sensorimotor system areas, including the precentral/postcentral gyrus, paracentral lobule (PCL) and temporal cortex compared to the EC condition from the four dimensions. The results consistently indicated that spontaneous brain activity is effectively related to EO and EC resting states, and the two resting states are of opposite brain activity in sensorimotor and occipital regions. It may provide new insight into the neural substrate of the resting state and help computational neuroscientists or neuropsychologists to choose an appropriate resting state condition to investigate various mental disorders from the resting state functional magnetic resonance imaging (fMRI) technique.

## Introduction

Studies have demonstrated that there are widespread significant differences in spontaneous brain activity between eyes-open (EO) and eyes-closed (EC) resting states using the functional magnetic resonance imaging (fMRI) technique. From the regional activity aspect of spontaneous brain activity, it was found that regional activity in the EO resting state was significantly higher in the bilateral middle occipital gyrus (MOG), orbital frontal cortex, right cuneus, fronto-parietal cortex and cerebellum regions, but significantly lower in the sensorimotor, visual, auditory, right paracentral lobule (PCL), retrosplenial cortex, insula, thalamus and cingulo-opercular regions compared to that in the EC resting state, by using the amplitude of low frequency fluctuation (ALFF; Yang et al., 2007; Yan et al., 2009; Liu et al., 2013; Zou et al., 2015; Qin et al., 2018), spectral density of the blood oxygenation level dependent signal (McAvoy et al., 2008, 2012), amplitude of spontaneous activity (Bianciardi et al., 2009; Zou et al., 2015), fractional ALFF (fALFF; Jao et al., 2013; Liang et al., 2014; Li Z. et al., 2016), and regional homogeneity (ReHo; Liu et al., 2013; Song et al., 2015) measures. From the regions synchronization aspect of spontaneous brain activity, it was found that functional connectivity in the EO resting state was significantly greater between the posterior cingulate cortex (PCC) and other brain areas, but significantly smaller between the whole thalamus and visual cortex, the PCC and the bilateral perisylvian regions, as compared to the EC resting state (Yan et al., 2009; Zou et al., 2009; Jao et al., 2013). From the network topological measures aspect of spontaneous brain activity, it was found that the nodal degree, average network connection distance, cliquishness and local efficiency distance in EO resting state were significantly increased, but global efficiency was significantly decreased as compared with the EC resting state (Jao et al., 2013; Xu et al., 2014). These significant differences of spontaneous brain activity between EO and EC resting states could support an “exteroceptive” and “interoceptive” mental states hypothesis (Marx et al., 2003), in which the “exteroceptive” mental state was characterized by attention and ocular motor activity during EO and the “interoceptive” mental state was characterized by imagination and multisensory activity during EC.

However, it is still largely unclear whether spontaneous brain activity is effectively related to EO and EC resting states. By contrast with group level significant analysis, it is necessary to establish the multivariable linking model that map spontaneous brain activity to the EO and EC resting states in getting the issue resolved. Using the support vector machine (SVM) classifier, one recent sturdy established the linking model that mapped the amplitude of spontaneous brain activity to EO and EC resting states, and found that fALFF in the sensorimotor regions could effectively related to these two resting states (Liang et al., 2014). The other important dimensions of spontaneous brain activity (Zuo and Xing, 2014; Aiello et al., 2015), including the ReHo (Zang et al., 2004), voxel-mirrored homotopic connectivity (VMHC; Zuo et al., 2010; Anderson et al., 2011), and degree centrality (DC; Buckner et al., 2009; Zuo et al., 2012) reflecting local or long distance functional connectivity, remain to be investigated and may provide new insight into the neural substrate of the resting state.

**Figure 1:**
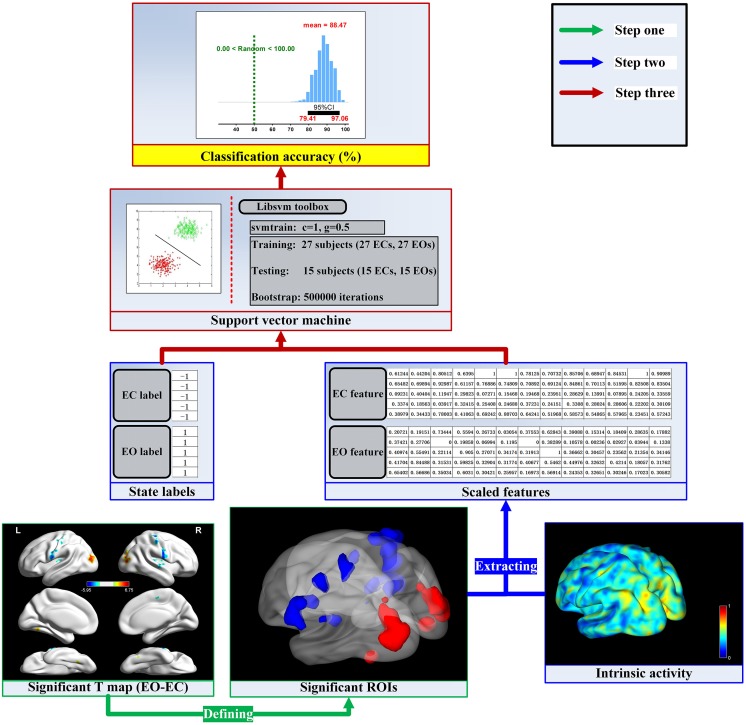
The flow chart of the analysis stream. For the given dimension (e.g., regional homogeneity, ReHo), the activity values were calculated in a voxel wise manner. The first step was to find the significant brain activity and then define the significant regions of interest (ROIs) by the paired *t*-test and Gaussian random field (GRF) correction method. The next step was to extract mean activity values across voxels in each ROI and scale them into a range of 0–1. Finally, we established the linking model and obtained the sampling distribution of the classification accuracy.

To further study the spontaneous brain activity related to the EO and EC resting states, we established the linking models for the fALFF, ReHo, VMHC and DC dimensions from machine learning perspective. The flow chart of the analysis stream for each dimension is shown in Figure 1. The fALFF, ReHo, VMHC and DC indices were suggested to represent the amplitude, local functional concordance and inter-hemisphere functional synchronization, and network centrality of spontaneous brain activity, respectively (Zang et al., 2004; Zou et al., 2008; Buckner et al., 2009; Zuo et al., 2010, 2012; Anderson et al., 2011; Zuo and Xing, 2014; Aiello et al., 2015). We extracted mean spontaneous brain activity as original features across significant cluster voxels which are defined by the paired *t*-test and Gaussian random field (GRF) correction method between the EO and EC resting states for each dimension of spontaneous brain activity. Applying the SVM and bootstrap technique, we established the linking models and obtained the sample distributions of classification accuracy for these linking models. The four linking models consistently indicated that spontaneous brain activity is effectively related to EO and EC resting states, and the two resting states are of opposite brain activity in the sensorimotor and occipital regions. It may provide new insight into the neural mechanisms of the resting state and help computational neuroscientists or neuropsychologists to choose an appropriate resting state condition to investigate various mental disorders from the resting state fMRI technique (Craddock et al., 2009; Iidaka, 2015; Kim et al., 2015; Rive et al., 2016; Suk et al., 2016; Billings et al., 2017; Khazaee et al., 2017; de Vos et al., 2018; Wang et al., 2018; Wei et al., 2018).

## Materials and Methods

### Eyes-Open/Eyes-Closed Dataset

The Eyes-open/Eyes-closed resting state fMRI dataset was shared by Liu et al. (2013) and can be freely download at http://fcon_1000.projects.nitrc.org/indi/retro/BeijingEOEC.html (Beijing: EO EC Study). The dataset included 48 healthy college students (aged 19–31 years, 24 female) from the Beijing Normal University in China. Each participant had one 3D T1-weighted image scan, three resting state fMRI scans, each of which consisted of 240 volumes and lasted for 8 min with TR = 2 s, and one 64-direction DTI scan. Participants were instructed to rest with their EC in the first resting state session. The second and third resting state sessions were counter-balanced with EO and EC conditions. In the EO condition, a blank screen with a black background was always presented. During all resting state sessions, the participants were requested to lie in the scanner quietly, not to fall asleep, and not to think about anything in particular. Immediately after each resting state session, the experiment operator spoke briefly with the participants. All the participants reported that they had not fallen asleep. The experiments were approved by the ethics committee of Institutional Review Board of Beijing Normal University Imaging Center for Brain Research. Written informed consent was obtained from each participant. In this study, we used the second and third resting state sessions.

### Data Preprocessing and Indices Computation

The standardized data preprocessing and indices computation of spontaneous brain activity were performed by clicking the “Run” button in the Data Processing Assistant for Resting-State fMRI (DPARSF *A*; Yan and Zang, 2010, Figure 2), which is based on the Statistical Parametric Mapping (SPM12^1^), and the toolbox for Data Processing and Analysis of Brain Imaging (DPABI; Yan et al., 2016).

**Figure 2:**
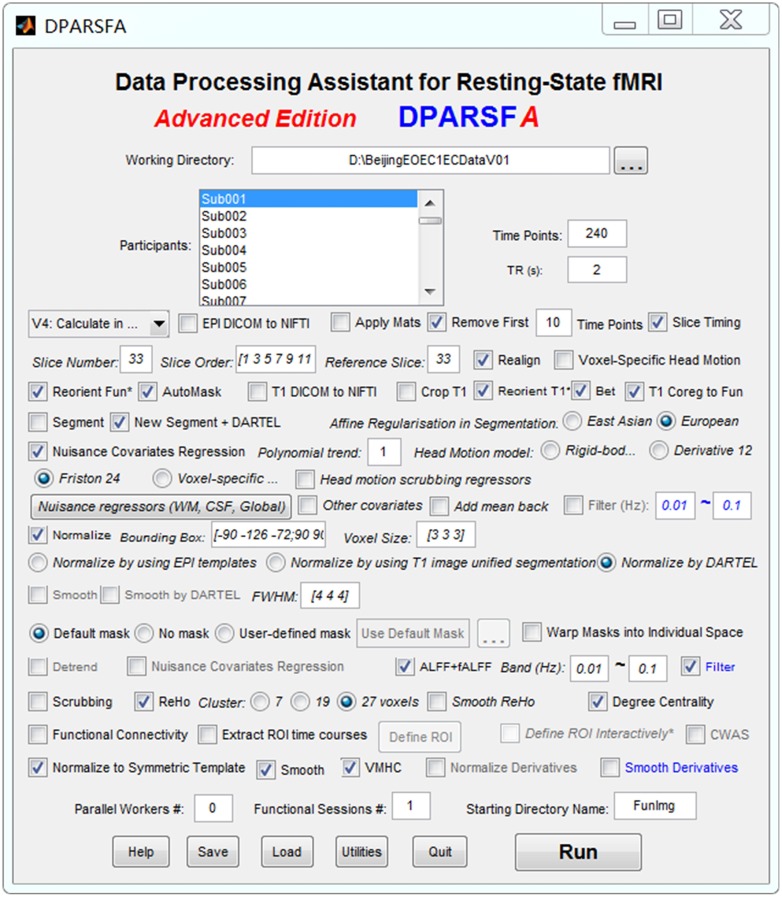
The setting panel for data preprocessing and indices computation.

The data preprocessing of EO and EC resting states was divided into two independent preprocessing runs. For each preprocessing run, the first 10 volumes were removed for scanner equilibration, leaving a total of 230 volumes to be used. The remaining functional volumes for each subject were corrected through the slice time step and realigned to estimate the six head motion parameters for head motion correction. Individual T1-weighted images were co-registered to the mean EPI image and then segmented into white matter (WM), gray matter, and cerebrospinal fluid (CSF) by the “New Segment + DARTEL” step (Ashburner, 2007). The linear trend, Friston 24-parameter and signals from WM and CSF were removed as nuisance variables to reduce head motion, respiratory and cardiac effects. Global signal regression was not performed because of concerns about increasing negative correlations (Murphy et al., 2009; Weissenbacher et al., 2009) and possible distortions (Gotts et al., 2013; Chi et al., 2016; Liu et al., 2017). After nuisance regression, the functional volumes were then normalized to MNI152 space in 3 × 3 × 3 mm^3^ voxel resolution by using the transformation information acquired from the previous DARTEL step (Figure 2).

The spontaneous brain activity indices in the present study were obtained as follows (Figure 2):

(1) ALFF (Zang et al., 2007) and fALLF (Zou et al., 2008). The ALFF for a voxel was the averaged amplitude of the square root power spectrum within a low frequency range (0.01–0.1 Hz) from a fast Fourier transformation of the time course. The fALFF for a voxel was the ratio of mean power spectrum of low-frequency range (0.01–0.1 Hz) to that of the entire frequency range. The ALFF and fALFF were suggested to reflect local spontaneous brain neuronal activity.
(2) ReHo (Zang et al., 2004), DC (Buckner et al., 2009; Zuo et al., 2012) and VMHC (Zuo et al., 2010; Anderson et al., 2011). The ReHo for a voxel was the Kendall’s coefficient of concordance between the time series of the voxel and those of its nearest neighbors (26-neighbor voxels), reflecting local functional concordance among fMRI time series (a certain local functional connectivity). The individual ReHo maps were converted with the Fisher’s z-transformation for normal distribution consideration. The DC for a voxel was the number of significant connections (*r* > 0.25, *P* < 0.05) in the voxel-based whole-brain functional connectivity map (Buckner et al., 2009), reflecting the network topological importance of that voxel. The VMHC for a voxel was the Pearson’s correlation coefficient between the time series of the voxel and that of its counterpart voxel at the same location in the opposite hemisphere, reflecting inter-hemisphere functional interaction. The individual VMHC maps were converted with the Fisher’s z-transformation.

The indices maps were all obtained in a voxel wise manner for the EO and EC resting states for each subject.

### Building the Linking Models

The linking models for the fALFF, ReHo, VMHC and DC dimensions were established respectively through the bootstrap technique and Libsvm toolbox (Chang and Lin, 2011,^2^). The flow chart of the analysis stream in the present work was shown in Figure 1.

The first step was to find the significant brain activity and then define the significant regions of interest (ROIs) at the group level. For a given dimension of spontaneous brain activity (e.g., ReHo), the paired sample *t*-test was employed to determine the T-statistic map by comparing EO and EC resting states. Head motion was controlled at the group level comparison by taking mean frame-wise displacement derived from Jenkinson’s formula as a covariate (Jenkinson et al., 2002; Satterthwaite et al., 2013; Yan et al., 2013). The T-statistic map was corrected by the GRF correction method with the 95% group mask and strict thresholds of *p* < 0.001 (voxel level) and *p* < 0.05 (cluster level), tow tailed (Chen et al., 2017). Finally, the significant ROIs mask was obtained by the corrected T-statistic map for the next feature extraction.

The second step was to extract spontaneous brain activity features and then label each feature vector as −1 or 1. For each voxel wised activity map in the EC resting state, we extracted mean activity values in each ROI of the above significant ROIs mask to from feature vector and then labeled the feature vector with label −1. For the EO resting state, we extracted feature vector as the EC condition do and labeled the feature vector with label 1. All features were scaled into a range of 0–1. The label and feature sets were prepared for establishing the linking models.

The third step was to obtain the linking model by the bootstrap technique and SVM classifier. To obtain the sampling distribution of the accuracy of the linking model, we employed bootstrap technique (Dougherty, 2014) with 63.20% subjects (about 27) used to train the SVM and 36.80% subjects (about 15) used to test the obtained SVM classifier with 500,000 repetitions for good generalizations. For each training and testing repetition, we randomly picked 27 subjects. Their labels and feature vectors were used to train SVM using the function “svmtrain” in the Libsvm toolbox to build the classification model, where we adopted the parameter *C* = 1 and the radial basis function kernel with gamma 0.5 for two classification problem (Chen et al., 2017). The feature vectors from the remaining 15 subjects were used to generate the corresponding predicted labels with function “svmpredict” in the Libsvm toolbox. The accuracy of the EC and EO classification were obtained by comparing the predicted labels with the real labels from the remaining 15 subjects (Dougherty, 2014). The linking model was the average of the 500,000 classification models, and the sampling distribution of the accuracy of the linking model was finally obtained from the sample accuracies of the 500,000 testing repetitions.

## Results

### Data Quality Assessment

A total of 42 subjects was used in the present study. We excluded three subjects whose brain is not covered by the resting state functional images. We also excluded three subjects because they are not right-handed subjects. For the remain subjects, their T1 and T2* images are of good quality, the 95% group mask represented well the total functional images, and none of the mean frame-wise displacement of head motion is larger than 0.2 mm.

### Different Spontaneous Brain Activity and Performance of the Linking Models

**Figure 3:**
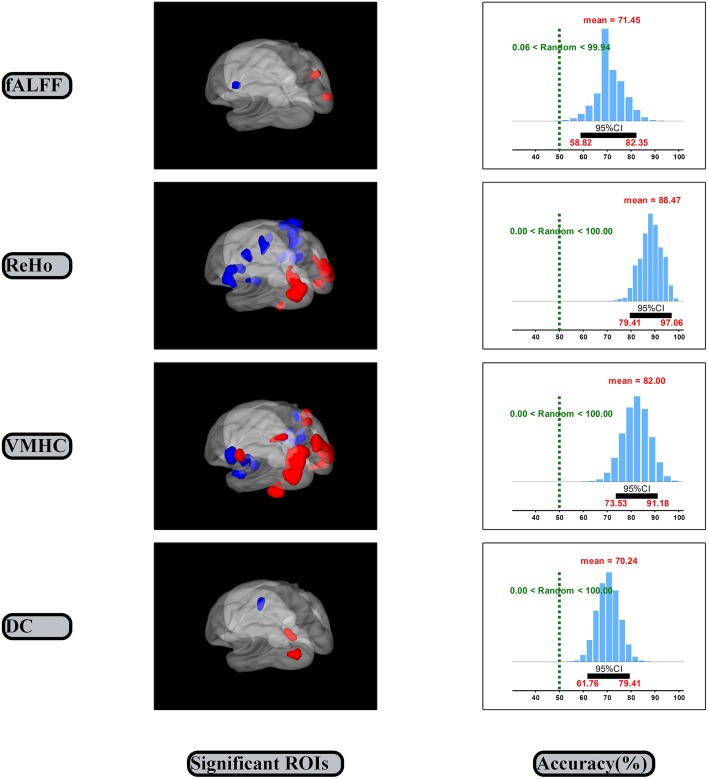
Significant ROIs and accuracies of linking models. CI, Confidence interval.

The SVM classification and bootstrap technique show that the sampling distributions of accuracy of the linking models are mainly located on the right side of the random performance dash line (the right side of the Figure 3). The classification accuracies are 0.71 (0.59, 0.82), 0.88 (0.79, 0.97), 0.82 (0.74, 0.91) and 0.70 (0.62, 0.79) for the fALFF, ReHo, VMHC and DC dimensions, respectively.

The significant spontaneous brain activity for each dimension was examined through means of the paired sample *t*-test and GRF multi-comparison correction by comparing EO and EC resting states (voxel-wise* p* = 0.001, *T* = 3.51, cluster-wise *p* = 0.05, tow tailed; see Table 1 and the left side of the Figure 3). Compared to the EC resting state, the fALFF values in the EO resting state are significantly increased in the right MOG and precuneus, but significantly decreased in the left precentral gyrus. The ReHo values in the EO resting state are significantly increased in the left fusiform gyrus and middle temporal gyrus, and right fusiform gyrus and MOG, but significantly decreased in the left putamen, postcentral gyrus, precentral gyrus and right superior temporal gyrus, insula, postcentral gyrus and PCL, relative to the EC resting state. Compared to the EC resting state, the VMHC values in the EO resting state are significantly greater in the bilateral MOG, postcentral gyrus and superior parietal gyrus, but significantly lower in the bilateral superior temporal gyrus, insula and postcentral gyrus. The DC values in the EO resting state are significantly larger in the left MOG and precuneus, but significantly smaller in the left precentral gyrus as compared with the EC resting state. In summary, the significant spontaneous brain activity areas were consistently located into sensorimotor and occipital attentional regions. Spontaneous brain activity in sensorimotor and occipital attentional regions was of relative uniform contributions for predicting EO and EC resting states (see Supplementary Figure S1).

**Table 1:** The change of spontaneous brain activity for the eyes-open (EO) vs. eyes-closed (EC) conditions.

Region	BA	No. voxels	Peak *t*-value	MNI		
				*X*	*Y*	*Z*
**fALFF**
R. middle occipital gyrus	−	4	4.30	33	−78	6
R. precuneus	−	4	5.10	27	−66	33
L. precentral gyrus	−	4	−4.27	−54	−15	36
**ReHo**
L. fusiform gyrus	19	33	5.03	−27	−60	−12
R. fusiform gyrus	37	19	4.62	39	−63	−15
R. superior temporal gyrus	22	39	−4.68	57	−12	0
L. middle temporal gyrus	19	195	6.55	−33	−81	15
R. insula	13	89	−5.41	36	−27	0
L. putamen	13	56	−4.80	−30	−15	6
R. postcentral gyrus	43	115	−5.95	66	−9	18
R. middle occipital gyrus	19	237	6.75	30	−87	18
L. postcentral gyrus	43	71	−5.15	−60	−3	18
R. postcentral gyrus	3	147	−5.75	45	−24	42
L. precentral gyrus	4	72	−5.18	−45	−15	54
R. paracentral lobule	6	12	−4.17	6	−15	45
L. precentral gyrus	4	62	−4.60	−24	−27	63
**VMHC**
Middle occipital gyrus	19	103	6.29	±45	−69	12
Middle occipital gyrus	19	385	7.92	±33	−84	9
Superior temporal gyrus	22	47	−5.17	±60	−21	0
Insula	41	54	−4.83	±45	−15	15
Postcentral gyrus	43	28	−4.68	±63	−9	21
Postcentral gyrus	1	18	4.61	±63	−24	36
Superior parietal gyrus	7	64	5.76	±24	−60	54
**DC**
L. middle occipital gyrus	19	43	5.80	−39	−81	15
L. precuneus	7	20	4.76	−21	−75	27
L. precentral gyrus	−	12	−3.81	−27	−27	60

## Discussion

The present study investigated the spontaneous brain activity patterns of the EO and EC resting states using multidimensional neuronal activity indices from machine learning perspective. The classification accuracies of the linking models are 0.71 (0.59, 0.82), 0.88 (0.79, 0.97), 0.82 (0.74, 0.91) and 0.70 (0.62, 0.79) for the fALFF, ReHo, VMHC and DC dimensions, respectively (Figure 3). Spontaneous brain activity in the EO resting state was significantly greater in the attentional systems areas, including the occipital, precuneus, fusiform and parietal cortex, but significantly lower in the sensorimotor areas, including the precentral/postcentral gyrus, PCL, putamen, insula and temporal cortex as compared with the EC resting state (Table 1).

The classification accuracies of the linking models could consistently indicate that spontaneous brain activity is effectively related to EO and EC resting states. The 95% confidence intervals of the fALFF, ReHo, VMHC and DC dimensions consistently exclude the random performance value 50% (Figure 3). It suggests that the correct prediction of the EO and EC resting states can be significantly better than random guesses from the each dimension of spontaneous brain activity. Further, the each dimension of spontaneous brain activity could well represent certain state-related aspect of spontaneous brain activity. The result of the fALFF dimension was consistent with the prior research (Liang et al., 2014). Liang et al. (2014) reported that the fALFF values in the sensorimotor regions could effectively related to the EO and EC resting states. We also obtained the accuracy 0.92 (0.89, 0.95) for the linking model that was established by combining features of the four dimensions (Dai et al., 2012; see Supplementary Figure S2). In line with the latest and best findings Zhou et al. (2018), reported that they obtained the high accuracy 0.95 by pair comparison between two within group conditions of resting state. In addition, spontaneous brain activity in sensorimotor and occipital attentional regions was of relative uniform contributions for predicting the two resting states (see Supplementary Figure S2). Hence, these valid linking models may consistently indicate that spontaneous brain activity might be effectively related to the EO and EC resting states.

The significant differences of spontaneous brain activity could consistently indicate that the EO and EC resting states are of opposite spontaneous brain activity in sensorimotor and occipital regions (Figure 3 and Table 1). Our results were mostly included into the results from the prior studies because we used the very strict statistical thresholds (voxel-wise* p* = 0.001, *T* = 3.51, cluster-wise *p* = 0.05, tow tailed; Chen et al., 2017).

From the fALFF dimension reflecting the intensity of local brain neuronal activity (Zang et al., 2007; Zou et al., 2008), we found that the fALFF values in the EO resting state are significantly increased in the right MOG and precuneus, but significantly decreased in the left precentral gyrus compared to the EC resting state. The results were consistent with prior researches (McAvoy et al., 2008; Bianciardi et al., 2009; Jao et al., 2013; Liu et al., 2013; Liang et al., 2014), which indicated that the amplitude of spontaneous brain activity were modulated by the EO and EC resting states. Jao et al. (2013) reported that the EO resting state was associated with decreased fALFF values mainly in the primary and secondary sensory cortical areas, the insula and the thalamus. Liu et al. (2013) observed significantly higher ALFF in areas including the bilateral MOG and orbital frontal cortex in the EO relative to the EC, and lower ALFF in regions including the motor network (e.g., the bilateral primary sensorimotor cortex, supplementary motor area and PCL), the auditory cortex and insula and thalamus etc. Liang et al. (2014) found that the fALFF values in EO were significantly increased in the fronto-parietal cortex, occipital cortex and cerebellum, but significantly decreased in the sensorimotor module and cingulo-opercular region compared those within EC. Together, the increased fALFF in the occipital regions (attentional system regions) may indicate that subjects increased attentional load or arousal, and the decreased fALFF in the sensorimotor regions might suggest that there was a suppression of sensory modalities during the EO resting state (Hüfner et al., 2009). This may support the “exteroceptive” and “interoceptive” mental states hypothesis (Marx et al., 2003) corresponding to the EO and EC resting states.

From the ReHo dimension reflecting local functional connectivity (Zang et al., 2004; Jiang and Zuo, 2016), we found that the ReHo values in the EO resting state are significantly increased in the left fusiform gyrus and middle temporal gyrus, and right fusiform gyrus and MOG, but significantly decreased in the left putamen, precentral/postcentral gyrus and right superior temporal gyrus, insula and PCL, relative to the EC resting state. In line with the findings of the fALFF dimension, increased ReHo values are mainly located in the attentional system regions, and decreased ReHo values are mainly located in sensorimotor regions. This might be due to the increased local neuronal activity in the attentional system regions and decreased local neuronal activity in the sensorimotor regions during the EO resting state. Hence, the local functional connectivity in terms of the ReHo similarly changed along with the changes of the fALFF corresponding to the EO and EC resting states. In addition, Liu et al. (2013) reported significantly increased ReHo values in the EO resting state in some regions in the visual cortex, including the bilateral MOG and right cuneus, and reduction of ReHo values within most parts of the motor network, auditory cortex, insula and right amygdala. Song et al. (2015) reported similar results that the ReHo values in the EO resting state was significantly decreased in the bilateral thalamus, supplementary motor area, sensorimotor cortex, superior temporal gyrus and insula, but significantly increased in the bilateral primary visual cortex, MOG and superior parietal gyrus compared to the EC resting state. Together, these results might indicate that the increased ReHo in the occipital regions (attentional system regions) were the representation of subjects’ increased attentional load or arousal, and the decreased ReHo in the sensorimotor regions were the representation of a suppression of sensory modalities during the EO resting state.

From the VMHC dimension reflecting inter-hemisphere functional connectivity (Zuo et al., 2010; Anderson et al., 2011), we found that the VMHC values in the EO resting state are significantly greater in the bilateral MOG, postcentral gyrus and superior parietal gyrus, but significantly lower in the bilateral superior temporal gyrus, insula and postcentral gyrus compared to the EC resting state. In line with the previous two dimensions (the fALFF and ReHo), increased VMHC values are mainly located in the attentional system regions, and decreased VMHC values are mainly located in sensorimotor regions. This might be due to brain functional areas located into two symmetric hemispheres that worked together to complete the increased attention and decreased sensory information processing during the EO resting state. Hence, the increased attentional load or arousal in the EO resting state may lead to increased functional connectivity in the occipital regions between the mirror pair voxels in the two hemispheres, and a suppression of sensory modalities in the EO resting state may lead to decreased functional connectivity in the sensorimotor regions between the mirror pair voxels in the two hemispheres.

From the DC dimension reflecting the network topological organization (Buckner et al., 2009; Zuo et al., 2012), we found that the DC values in the EO resting state are significantly larger in the left MOG and precuneus, but significantly smaller in the left precentral gyrus as compared with the EC resting state. In line with the previous three dimensions (the fALFF, ReHo and VMHC), increased DC values are mainly located in the attentional system regions, and decreased DC values are mainly located in the sensorimotor regions. The functional connectivity between the attentional system regions and the other brain areas were enhanced to allocate attention resource toward the environment during the EO resting state. By contrast, the functional connectivity between the sensorimotor regions and the other brain areas were weakened to suppress the interoceptive sensorimotor information processing during the EO resting state. These results were in line with the previous studies (Xu et al., 2014), which indicated that the nodal degrees in the EO resting state were higher in the “exteroceptive” network, including the attentional system (e.g., superior parietal gyrus and inferior parietal lobule), but lower in the “interoceptive” network, including the somatosensory system (e.g., postcentral gyrus), relative to the EC resting state.

Summarizing, the results from the four dimensions consistently indicated that spontaneous brain activity in the EO resting state would be higher in the occipital regions, particularly in the attentional system regions, but lower in the sensorimotor regions compared to the EC resting state. Opposite brain activity in sensorimotor and occipital regions may add more new understanding into the neural basis of the EO and EC resting states, and may further confirm an “exteroceptive” and “interoceptive” mental states hypothesis (Marx et al., 2003).

## Limitations

The present study mainly contains the three limitations. First, we employed the most widely used machine learning approach (SVM classifier) to establish the linking models for spontaneous brain activity. Many other machine learning approaches (such as, the logistical regression, Fisher discriminative analysis, artificial neural network and convolutional neural network, etc.; Dai et al., 2012; Wei et al., 2016; Dimitriadis and Salis, 2017) should be also considered in the latter studies to confirm the effectiveness of the different linking models. Second, we investigated the spontaneous brain activity patterns related to the EO and EC resting states for the healthy subjects. In fact, the resting state fMRI technique was most widely used in investigating various mental disorders by requesting all subjects in the EO or EC resting state. It is still unknown whether resting state condition with EO or EC is a confounding factor for the comparison between the diseases and healthy controls (Alba et al., 2016; Janssen et al., 2016; Li W. et al., 2016; Kan et al., 2017; Nair et al., 2017). Future studies using resting state paradigm should clearly indicate whether they are using data with EO or EC. Thirdly, it may try more public datasets and test whether the findings are sensitivity to different datasets if we can elegantly solve the possible heterogeneous problems of different datasets.

## Conclusion

In the present study, we further clarify the spontaneous brain activity mechanisms of the EO and EC resting states using multidimensional evidences from machine learning perspective based on the public Eyes-open/Eyes-closed dataset. The results consistently indicated that spontaneous brain activity is effectively related to EO and EC resting states, and these two resting states are of opposite spontaneous brain activity in sensorimotor and occipital regions. It may provide new insight into the neural basis of the resting state and help computational neuroscientists or neuropsychologists to choose an appropriate resting state condition to investigate various mental disorders from the resting state fMRI technique.

## Author Contributions

CL and GL: providing the ideas. JW: establishing the linking models and obtaining the spontaneous brain activity patterns. TC, JQ and DW: drawing the pictures. All authors wrote and reviewed the manuscript.

## Conflict of Interest Statement

The authors declare that the research was conducted in the absence of any commercial or financial relationships that could be construed as a potential conflict of interest. The reviewer LJ declared a shared affiliation, though no other collaboration, with one of the authors TC to the handling Editor.

## Footnotes

^1^http://www.fil.ion.ucl.ac.uk/spm

^2^https://www.csie.ntu.edu.tw/~cjlin/libsvm/

## Supplementary Material

FIGURE S1 Significant ROIs and contributions for predicting EO and EC resting states. Spontaneous brain activity in sensorimotor and occipital attentional regions was of relative uniform contributions for predicting the two resting states.

FIGURE S2 Accuracy and contribution of cascading features of the four dimensions.
