# Supplementary figures and images for: Eyes-Open and Eyes-Closed Resting States With Opposite Brain Activity in Sensorimotor and Occipital Regions: Multidimensional Evidences From Machine Learning Perspective
Source: Front Hum Neurosci. 2018 Oct 18;12:422. doi: 10.3389/fnhum.2018.00422 (PMC6200849; doi:10.3389/fnhum.2018.00422)

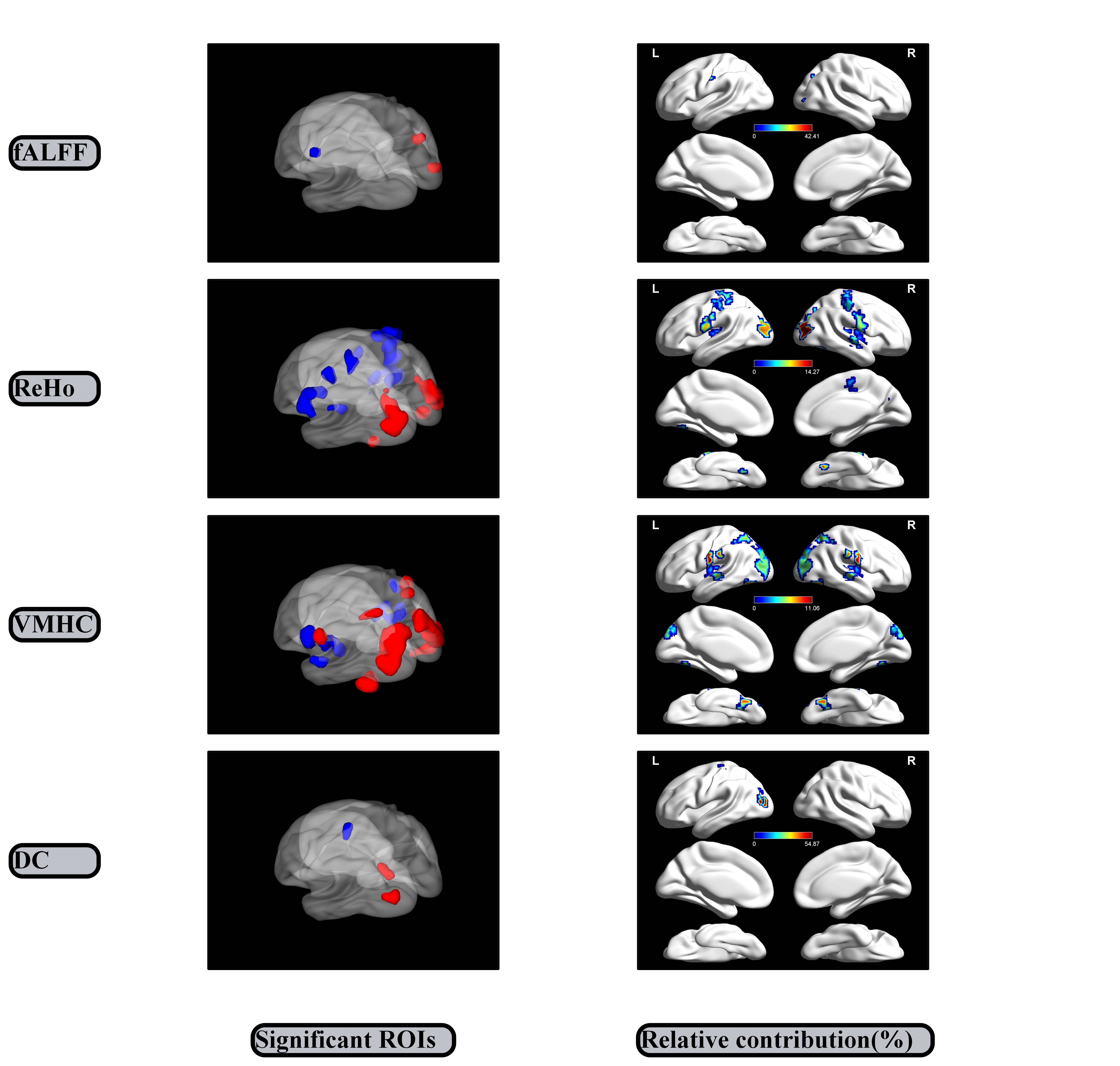

Supplement: FIGURE S1 — Significant ROIs and contributions for predicting EO and EC resting states. Spontaneous brain activity in sensorimotor and occipital attentional regions was of relative uniform contributions for predicting the two resting states. [file Image_1.jpg]

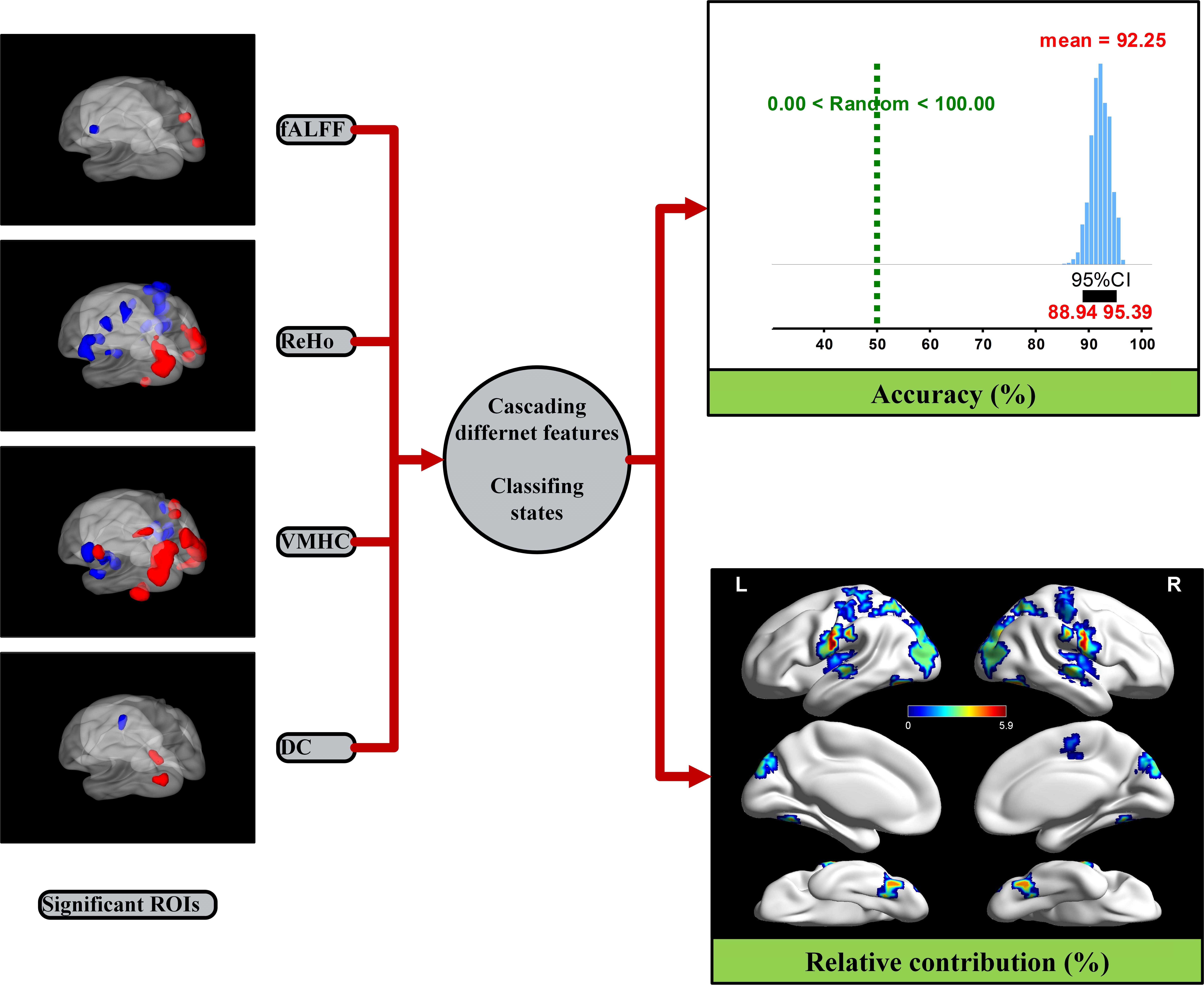

Supplement: FIGURE S2 — Accuracy and contribution of cascading features of the four dimensions. [file Image_2.jpg]
